# An efficient sorghum transformation system using embryogenic calli derived from mature seeds

**DOI:** 10.7717/peerj.11849

**Published:** 2021-08-05

**Authors:** Lihua Wang, Li Gao, Guoquan Liu, Ruirui Meng, Yanlong Liu, Jieqin Li

**Affiliations:** 1 College of Agriculture, Anhui Science and Technology University, Fengyang, China; 2 Queensland Alliance for Agriculture and Food Innovation, The University of Queensland, Brisbane, Australia

**Keywords:** Sorghum, Particle bombardment, Genetic transformation, Transgenic plants, Mature seeds

## Abstract

Significant progress has been made on sorghum transformation in the last decades; however, the transformation process has been constrained by the availability of immature embryos because most of the researchers have utilized immature embryos as favorable explants. Although immature embryos have been proven to be optimal for tissue culture and transformation, isolation of immature embryos is time-consuming, labor-intensive, and limited by warm weather. In this study, we developed an efficient genetic transformation system using mature seeds as explants. The *nptII* and *gus* gene, used as the selective marker and report gene respectively, have been co-transformed by particle bombardment. After optimization of tissue culture, the G418 concentration, and transgenic, the average transformation frequency at 13.33% was achieved routinely. The transgenic events and transgene copy numbers were determined by PCR and RT-PCR, respectively. The geneticin selection and GUS staining on T_1_ seedlings confirmed that the transgenic plants were heritable. Our results demonstrated that the efficient sorghum transformation system has been established using mature seeds as explants. This transformation system will promote sorghum research on genetic engineering and genome editing without seasonal weather conditions restriction and explant resources restriction.

## Introduction

Sorghum (*Sorghum bicolor*) is the fifth most cultivated cereal crops in the world. It is a multipurpose crop that can be used as food, forage, biofuel, and industrial materials (De Morais Cardoso et al., 2017; Dahlberg, 2019). In Africa and parts of Asia, sorghum is used as a staple food (Adebo, 2020). With the availability of sorghum genome sequencing, more and more researchers have focused on the manipulation of gene function in sorghum because sorghum has the smallest genome size of the agronomically important C_4_ crops, and further, sorghum has been studied for its use as a C_4_ model crop (Paterson et al., 2009; Mace et al., 2013).

Sorghum has been widely recognized as a recalcitrant crop in terms of tissue culture and transformation since the first transgenic sorghum was reported in 1993 (Casas et al., 1993). The transformation efficiency was less than 0.1% using a particle delivery system approach (Casas et al., 1993). Since then, remarkable progress has been made on sorghum tissue culture, transformation, and genome editing (Liu & Godwin, 2012; Liu, Gilding & Godwin, 2015; Belide et al., 2017; Che et al., 2018; Liu et al., 2020). The transformation efficiencies have been significantly improved to around 30% in both *Agrobacterium tumefaciens* (*Agrobacterium*)-mediated and particle bombardment transformation systems in recent years.

Amongst reported sorghum studies on genetic transformation, immature embryos were initiated as favorable explants. However, isolation of immature embryos are costly and laborious. Besides, it requires the continued planting of stock plants in a temperature-controlled greenhouse to ensure constant access to a supply of immature embryos (Belide et al., 2017). Furthermore, sorghum is a summer crop and prefers warm weather. The transformation efficiency using immature embryos is signficnatly lower in winter than summer because the physiological condition of the donor plant has a significant impact on the subsequent efficiency of the tissue culture process (Do et al., 2016). In contrast, mature seeds provide an ideal solution of the impediments presented by immature embryos because mature seeds are independent of seasonal effects, are convenient to access, and have straightforward storage requirements. Therefore, there is a growing demand to exploit an alternate approach to the use of immature embryos as explants.

Previously, we screened 120 sorghum accessions and established a highly efficient tissue culture system using mature seeds (Li et al., 2017), which has paved the way for genetic transformation. In the present study, we developed an efficient transformation protocol using mature seeds as explants. We also analyzed the molecular characterization of transgenic plants in T_0_ and T_1_ generation to confirm both the presence and inheritance of the introduced transgenes.

## Material and Methods

### Plant material

The mature seeds of sorghum accession IS4698, which we reported previously to perform best in tissue culture (Li et al., 2017), were used for the tissue culture process and subsequent transformation procedure. Only healthy seeds were selected and sterilized *via* being saturated and shaken in 70% alcohol for 5 min. The seeds were then transferred to 0.1% (*v/v*) mercury chloride (HgCl_2_) solution which contained 2–3 drops of Tween 20, and were shaken for 15 min at room temperature. Subsequently, seeds were washed with five changes of sterilized water. Finally, the seeds were placed on sterilized paper towel for 10 min and were then transferred to the induction medium.

### Plasmids

**Figure 1:**
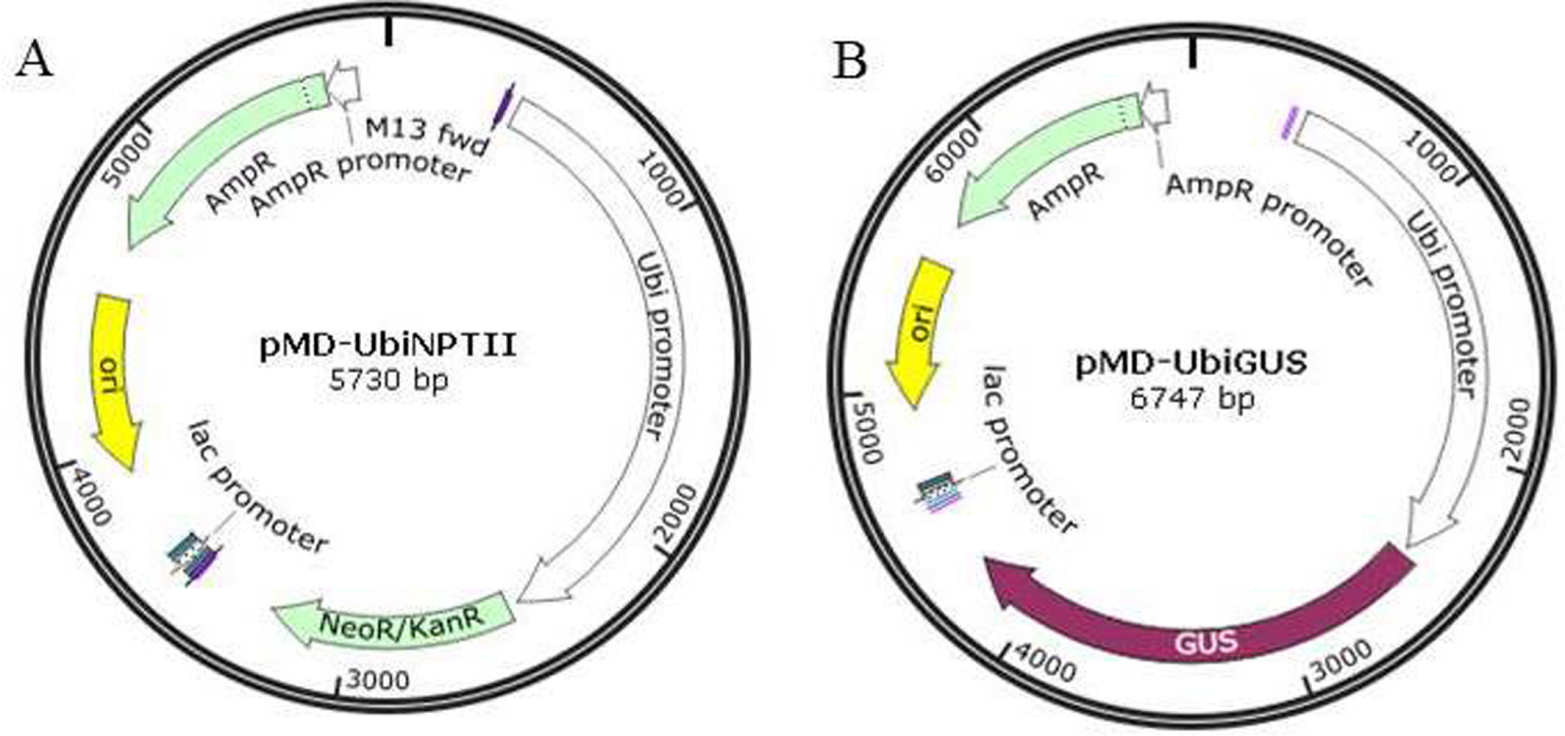
The plasmid maps for transformation. (A) pMD-UbiNPTII. (B) pMD-UbiGUS.

Two plasmids, pMD18T-UBI-NPTII and pMD18T-UBI-GUS were designed and cloned using the pMD18-T vector (Takara, Japan). The expression of both the *NPTII* and *GUS* genes was driven by the maize ubiquitin (*ubi*) promoter, and each of these two coding sequences were terminated with the octopine synthase terminator (OCS) of *A. tumefaciens* (Fig. 1).

### Media for tissue culture

All media were based on MS medium which contains 4.43 g/L MS powder with vitamins (PhytoTchnology Laboratories), 30 g/L sucrose, and 3.5 g/L phytagel. The pH of all media was adjusted to 5.7 before autoclaving at 121 °C for 15 min. The plant growth regulators (as stated below for each media type) were filter-sterilized and added after autoclaving.

Callus induction medium: MS medium supplemented with 1.0 mg/L KH_2_PO_4,_ 0.1 g/L Inositol, 1.0 g/L Aspartic acid,1.0 g/L proline, 0.5 mg/L Nicotinic acid, 1.875 mg/L CuSO _4,_0.4 mg/L Glycine, 0.5 mg/L Vitamin B6, 0.1 mg/L Thiamin, 10 mg/L Ascorbic acid, and 5.0 mg/L 2,4-D.

Subculture medium: MS medium supplemented with 1.0 mg/L KH_2_PO_4,_ 0.1 g/L Inositol, 1.0 g/L Aspartic acid,1.0 g/L proline, 0.5 mg/L Nicotinic acid, 1.875 mg/L CuSO_4,_ 0.4 mg/L Glycine, 0.5 mg/L Vitamin B6, 0.1 mg/L Thiamin, 10 mg/L Ascorbic acid, and 2.0 mg/L 2,4-D.

Selective regeneration medium: MS medium supplemented with 2.0 mg/L 6-Benzylaminopurine, 1.0 mg/L Indole-3-acetic acid, and 30 mg/L G418.

Rooting medium: 1/2 strength MS medium supplemented with 2.0 mg/L 6-Benzylaminopurine, 1.0 mg/L Indole-3-acetic acid, 1.0 mg/L 1-naphthylacetic acid, and 30 mg/L G418.

Osmotic medium: MS medium supplemented with 37.56 g/L sorbitol and 37.17 g/L mannitol.

### Growth conditions for tissue culture

The temperature in the tissue culture room was set at 28 °C for callus induction, subculture, regeneration, and rooting. Tissues were kept in dark for callus induction and subsequent phases of subculture. The plant tissues were then kept under the lighting regime of 16 h light (100–150 µmol m^−2^ s^−1^) and 8 h dark for aerial tissue regeneration and root induction.

### Kill curve of calli derived from mature seeds

A kill curve experiment was employed using calli derived from mature seeds. All calli produced from one seed was counted as one unit two weeks after initiation. Treatment with different selection concentrations, including 0, 10, 20, 30, and 40 mg/L G418, were prepared in selection regeneration medium. A total of 20 units of calli was subcultured fortnightly onto selection regeneration medium and placed on shelves in the tissue culture room. Four weeks later, the numbers of calli generating shoots were recorded.

### Optimization of DNA delivery parameters

To optimize DNA delivery parameters of the Bio-Rad PDS-1000/He System, two sizes of gold particles (0.6 or 1 µm), two Helium pressures (900 or 1100 psi), and two target distances from the stopping mesh to the calli (9 or 12 cm) were applied in this experiment. A total of eight combinations of different parameters were assessed (Table 1). As for each combination, 27 calli were tested in triplicate. The GUS staining was performed by placing the transformed calli, and the control calli, into GUS staining solution three days post-bombardment, with the calli incubated in the staining solution for 24 h (h) at 37 °C. Post this incubation period, the number of callus with blue foci was recorded.

**Table 1:** The combination of DNA delivery parameters.

Parameters	1	2	3	4	5	6	7	8
Pressure (psi)	1100	1100	1100	1100	900	900	900	900
Target Distance (cm)	9	12	9	12	9	12	9	12
The diameter of the gold particle (µm)	0.6	0.6	1	1	0.6	0.6	1	1

### Transformation protocol

Calli were used for particle transformation 14 days after induction. The transgenic protocol was performed as described previously by Liu & Godwin (2012) with some modification. Briefly, the transformation parameters were optimized for calli derived from mature seeds as detailed in Table 1. Equal amount of the plasmids pMD18T-UBI-GUS and pMD-UbiNPTII were mixed for co-transformation, namely 5 µL of each plasmid at a concentrated of 1.0 µg/µL were mixed together. The bombarded calli were kept on osmotic medium for 3–4 h, then the calli were transferred onto subculture medium and kept in dark for 3 days.

The calli on subculture medium were transferred onto selection regeneration medium. Then calli were continuously subcultured on selection regeneration medium fortnightly. Once shoot material had emerged from the calli and elongated to a length of 5 to 8 cm, the plantlet-like material was transferred onto selective rooting medium. When plantlets developed sufficient roots (generally >10 roots at a length of >one cm), they were transferred into pots containing TS1 soil (KLASMANN, Germany), and were transferred to the glasshouse.

### PCR screening of *in planta* transformation events

Genomic DNA was extracted from young leaves of putative transgenic lines and non-transgenic line IS4698 using a DNAsecure Plant kit (Tiangen, China), according to the manufacture’s instructions. Primers specific to the *NPTII* and *GUS* coding sequences were designed to confirm the presence of *NPTII* and *GUS* transgenes, respectively. The sequences of the primers used for this analysis were, NPTIINN-f TCCGGTGCCCTGAATGAA and NPTIINN-r GTCGATGAATCCAGAAAAGC, and GUS-f AGCGTTGAACTGCGTGAT and GUS-r GTTCTTTCGGCTTGTTGC. A 15 µL PCR reaction mixture was composed of 1.5 µL DNA (50 ng/µL), 7.5 µL 2 X KOD buffer, 2.7 µL primers (2 µM), 3.0 µL dNTPs (2.0 mM), 0.3 µL KOD (1.0 U/µL). The PCR procedure was as follows; 1 cycle of initial denaturation at 94 °C for 5 min, followed by 34 cycles of 95 °C for 30 s, 50 °C for 30 s, 68 °C for 40 s, and a final elongation step at 68 °C for 5 min. The amplified PCR products were separated on 1.0% (*w/v*) agarose gels.

### RT-PCR to determine *nptII* gene copy number

Transgene copy number was calculated as described by Casu and colleagues (2012). A total of 12 µL of RT-PCR reaction mix composed of 1.0 µL genomic DNA (1.0 ng/µL), 6.0 µL SYBR Green Master Mix Reagent (Applied Biosystems) and 0.6 pMol each of gene-specific forward and reverse primer. The PCR procedure used was as follows; 1 × 50 ° C for 2 min and 95 °C for 10 min, followed by 40 cycles of 95 °C for 15 s and 60 °C for 1 min. The RT-PCR was performed on an ABI prism 7900 Real-Time PCR System.

Anthranilate phosphoribosyltransferase (APRT, Sobic.002G303300) and thiosulfate sulfurtransferase (TST, Sobic.008G162000) were selected as reference genes. The copy number of the *nptII* gene was calculated based on the method of Casu, Selivanova & Perroux (2012). A gene copy number index for each sample was obtained by using the following formula: gene copy number index = PCR efficiency from the reference/PCR efficiency from the test gene. PCR efficiency was calculated using LinRegPCR software (Ramakers et al., 2003). The primers used for this analysis were as follows; APRT-f, TGACACATTCCCAACCTCAA and APRT-r ATCTCTCTCCCTGAGTGGCA, and; TST-f ACATGCTGCCATCTGAAAAG together with TST-r CAGCCCCTTTCCATCATAAA, and for the transgene, the primers NPTIIq-f GCCGAATATCATGGTGGAA and NPTII-r AATATCACGGGTAGCCAACG were used.

### Inheritance of the introduced transgene

Seeds of T_1_ lines were selected for inheritance analysis. Seeds of the non-transgenic plant line, IS4698, were used as the control. The seeds of each plant line were placed on a Petri dish that contained two filter papers. The seeds were partially saturated with sterilized water and kept in the dark for 3 days at 28 °C for germination. The germinated seedlings were then saturated in sterilized water that contained 30 mg/L G418. The plates were put on the shelf in the tissue cuture room under the lighting conditions of 16 h light and 8 h dark at 28 °C for 1 week. The vigor of transgenic seedlings was recorded for comparison to the non-transgenic control.

### GUS expression analysis of the T_1_ generation

The T_1_ seedlings, panicles, and stems were assessed *via* GUS staining. The samples were put into GUS staining buffer that contained 1.0 mg/mL freshly dissolved X-Gluc and were vacuum infiltrated for 1 h. After staining for 48 h at 37 °C, tissues were washed *via* treatment with 75% ethanol for 48 h until the chlorophyll was completely removed. Then the seedlings, panicles, and stems were photographed with a NIKON D3200 (Japan) camera. The cross-section of stems was taken with the use of a Leica DM3000 (Wetziar, Germany) microscope.

## Results

### The kill curve of G418 concentrations on callus

The calli were put on the regeneration medium containing different G418 concentrations (Table 2). The five different G418 concentrations were used to screen the appropriate killing concentration for calli. After four weeks, no shoots were regenerated on calli cultivated on media with G418 concentrations of 30 and 40 mg/L. However, shoots were regenerated on calli grown on medium supplemented with G418 concentration of 10 and 20 mg/L. These calli did not grow well and displayed the effects of growth on G418 selection medium. By comparison, all calli survived on regeneration medium without selection G418 and regenerated the most shoot material (Table 2). Our results displayed that 30 mg/L G418 was sufficient enough to prevent the calli from forming shoots. Hence, a concentration of 30 mg/L G418 was employed as the selection concentration for the generated IS4698 transformant lines.

**Table 2:** The kill curve of callus in 4 weeks.

G418 concentrations	Callus with shoots	Survival callus
0	8	20
10	4	16
20	2	10
30	0	5
40	0	3

### Optimization of DNA delivery parameters

The different parameters for DNA delivery were tested to identify the optimal combination. The data were statically analyzed by ANOVA. The results showed that eight treatments are significantly different (Table 3). Among the eight treatments, transient gene expression efficiency is significant higher in the treatment 2 and 5 than the rest of treatments. The highest transient gene expression efficiency was 92.56% obtained from treatment 2, of which contained parameters of (1) 0.6 µm gold particles, (2) a 12 cm target distance, and (3) 1,100 psi helium pressure. Therefore, these DNA delivery parameters were applied in all of our following experimentation.

**Table 3:** The GUS staining of calli on transient gene expression.

Treatments	Percentage of staining (%)
2	92.59 a
5	90.74 a
1	65.17 b
8	58.00 b
3	54.90 b
7	28.89 c
4	26.19 c
6	23.45 c

**Notes.**

a, b, c, and d indicates significant at 0.05 levels (*p* ¡ 0.05).

### Transformation efficiency of mature seeds

**Figure 2:**
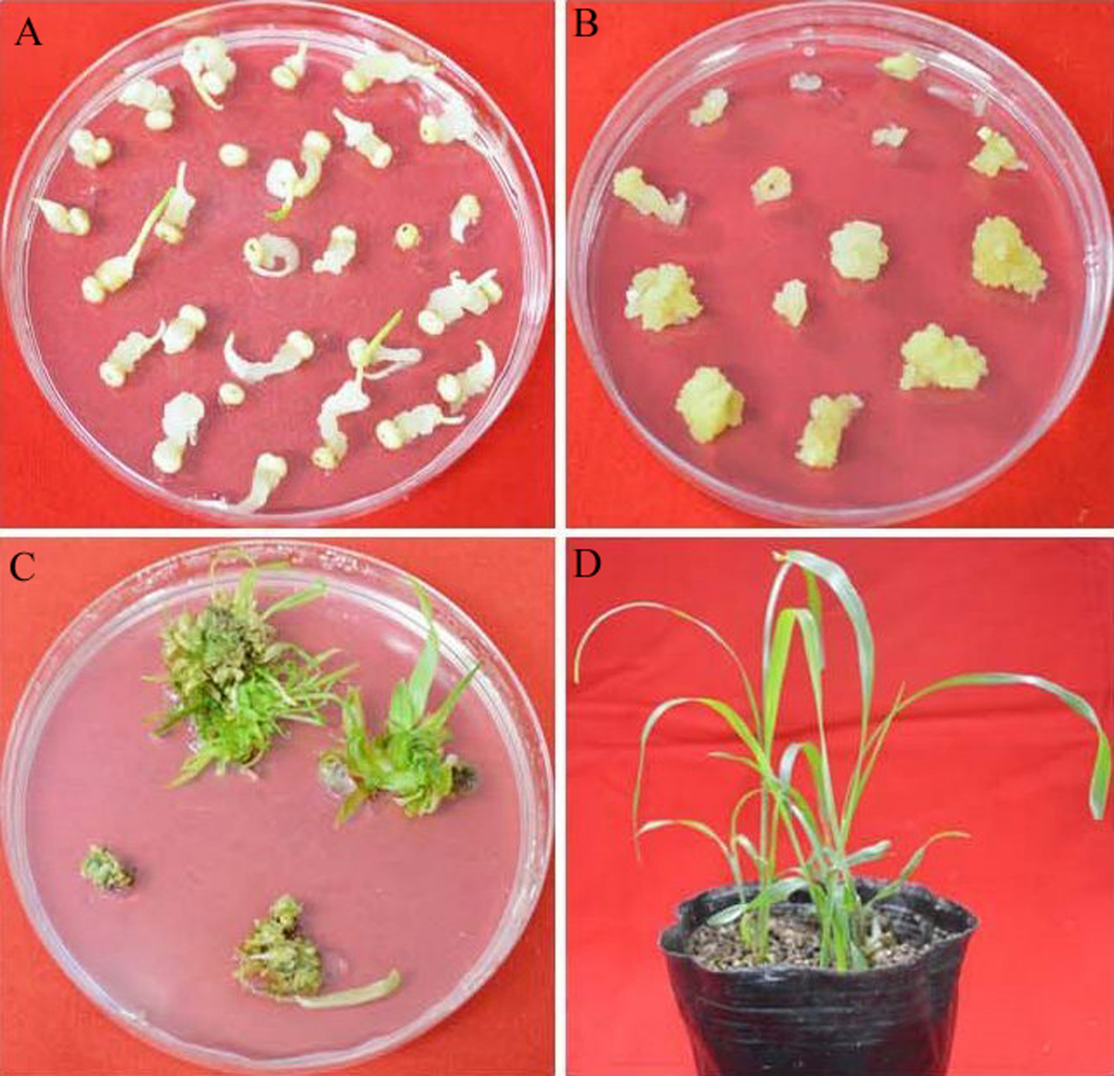
The sorghum transformation system using mature seeds. (A) Calli derived from mature seeds on the induction medium. (B) Calli on the subculture medium. (C) Shoots on the selection regeneration medium. (D) Putative transgenic plants in the pot.

The white and transparent callus were induced from sorghum IS4698 mature seeds two weeks after initiation (Fig. 2A). Then, only white and transparent calli were selected and transferred onto the subculture medium. Two weeks later, the compact and yellowish embryogenic calli were produced from the white and transparent calli (Fig. 2B). The embryogenic calli were selected as the material to perform transformation. The transformed calli were placed on the selection regeneration medium post-bombardment for 4–6 weeks. Most of the calli did not survive the four week period, but some calli did regenerate shoots (Fig. 2C). The shoots were transferred to rooting medium for 2 to 3 weeks. After roots became well-developed, the plantlets were planted in a pot for 10 days (Fig. 2D), and then the pots were transferred to the greenhouse. The whole process took approximately 11 to 15 weeks from callus induction to transgenic plant obtainment.

In three independent experiments, a total of 142 calli, were utilized for co-transformation with the plasmid-based pMD18T-UBI-GUS and pMD-UbiNPTII plant expression vectors. A total of 19 independent transgenic plants were recovered from these experiments, and across the three experiments, transformation efficiencies varied from 10.00% to 18.75%, to give an average transformation frequency of 13.33% (Table 4).

**Table 4:** Transformation efficiency of sorghum IS4698 mature seeds.

Experiment	Bombarded Callus	Transgenic events	Transformation efficiency
1	32	6	18.75%
2	40	4	10%
3	70	9	12.85%
Total	142	19	13.33%

### PCR screening of putatively transformed plants

**Figure 3:**
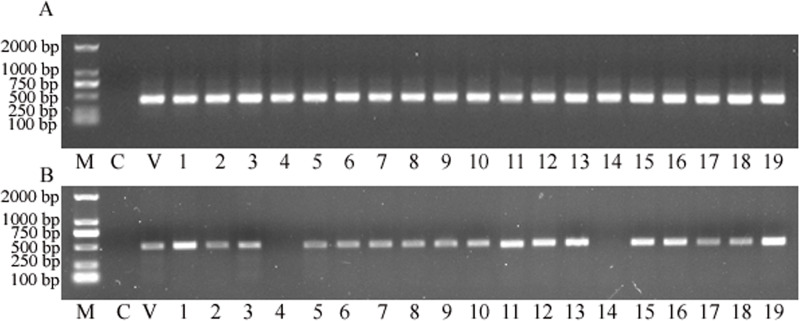
The PCR screening for *nptII* and *gus* genes. (A) Amplification for the NPTII gene. (B) Amplification for the *gus* gene. M, D2000 (Tiangen, China); C, control sample, V, positive control vector, numbers 1 to 19 are putative transgenic samples.

Total genomic DNA was extracted from transformed plants and then PCR detection was deployed to identify whether the plant expression vector delivered transgenes were successfully transformed into the sorghum genome. The results showed that the targeted *nptII* gene was amplified from all putative transgenic plants, and with no *nptII*-specific PCR product amplified from the control (Fig. 3A). This analysis strongly suggested that all 19 plants obtained from the tissue culture process were indeed transgenic. Subsequently, the *gus* gene was targeted for PCR amplification. The analysis revealed that 17 of the 19 samples retruned an amplification product specific to the *gus* gene (Fig. 3B), to reveal a co-transformation rate of 89.47%.

### RT-PCR to determine *nptII* gene copy number

The transgene copy number was estimated using RT-PCR analysis of genomic DNA from the 19 T_0_ lines. The primers of the *nptII* gene were used to estimate the gene copy number. The primers specific to two reference genes, APRT and TST, were also used in this analysis. Correlation analysis displayed similar patterns of gene copy numbers of transgenic samples (*R* = 0.99) when either APRT or TST was used as the reference gene. Therefore, no significant difference (*t* = −0.07, *P* = 0.94) between these two reference genes was identified. The estimated results demonstrated that 21% of the generated transformant lines had a single transgene copy, 53% of lines harboured between 2 to 4 transgene copies, and 26% of the analysed lines were determined to have more than 4 copies of the inserted *nptII* transgene (Table 5).

**Table 5:** RT-PCR for estimating *nptII* gene copy number of transgenic plants.

Line type	Estimated NPTII gene copy number		Line type	Estimated NPTII gene copy number	
	APRT	TST		APRT	TST
Wild type line 1	<1	<1	NPTII-10	6	6
Wild type line 2	<1	<1	NPTII-11	1	1
NPTII-1	2	2	NPTII-12	3	3
NPTII-2	1	1	NPTII-13	1	1
NPTII-3	6	6	NPTII-14	2	2
NPTII-4	2	2	NPTII-15	4	4
NPTII-5	7	7	NPTII-16	3	3
NPTII-6	4	4	NPTII-17	2	2
NPTII-7	3	3	NPTII-18	8	8
NPTII-8	7	7	NPTII-19	2	2
NPTII-9	1	1			

### Transgene inheritance in the T_1_ generation

**Figure 4:**
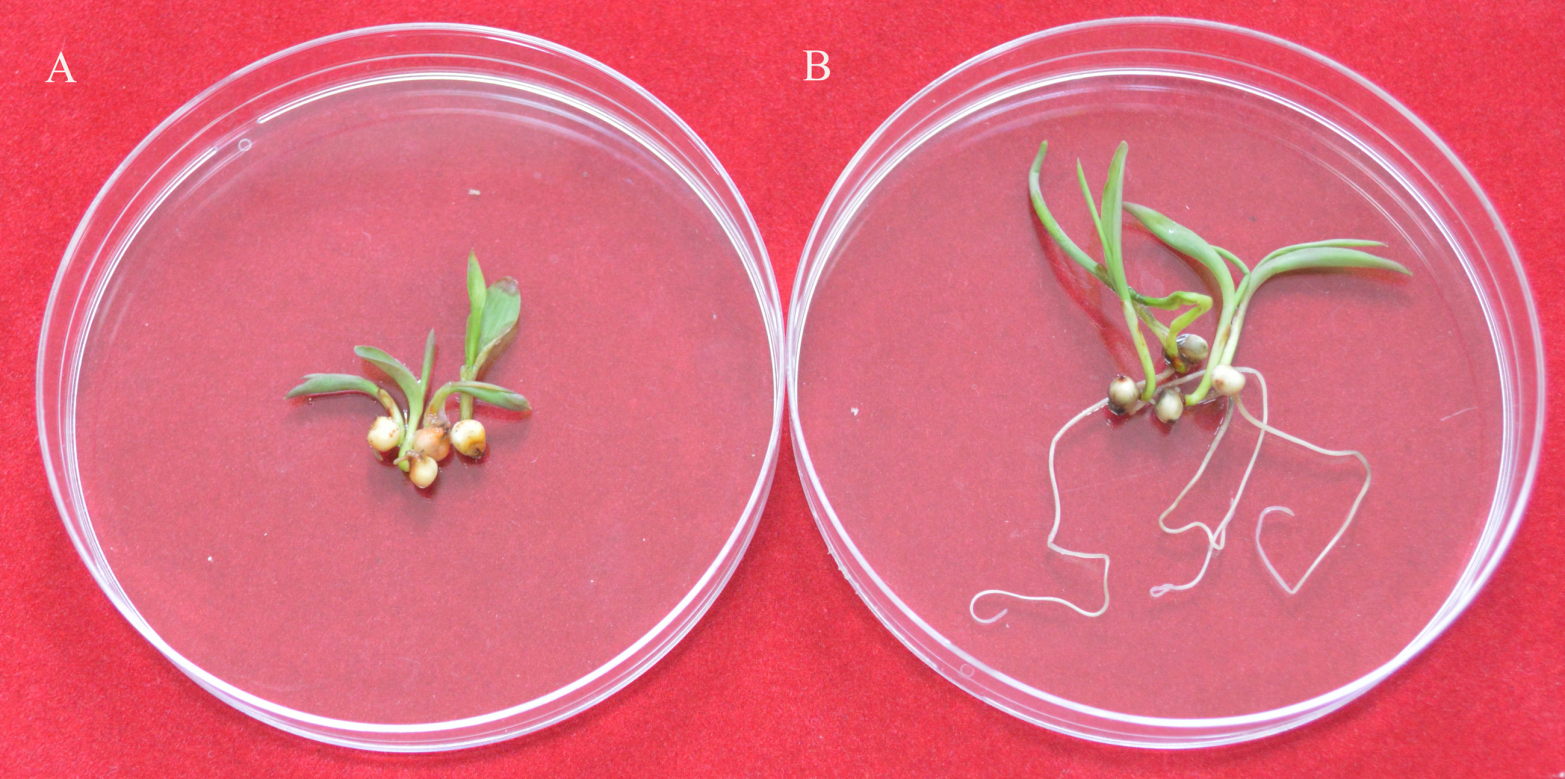
The G418 resistant assay. (A) The non-transgenic seedlings. (B) The transgenic T_1_ seedlings.

To identify the expression of the *nptII* gene in the T_1_ generation, the transgenic seeds were germinated under G418 selection (30 mg/L). The results showed the null segregants of the *nptII* gene and non-transgenic seedlings did not grow roots on the G418 selection medium (Fig. 4A). In contrast, T_1_ seedlings with the *nptII* gene grew normally and developed a vigorous root system (Fig. 4B). This analysis showed that the introduced transgene is stably inherited by the next generation.

### GUS expression analysis of the T_1_ generation

**Figure 5:**
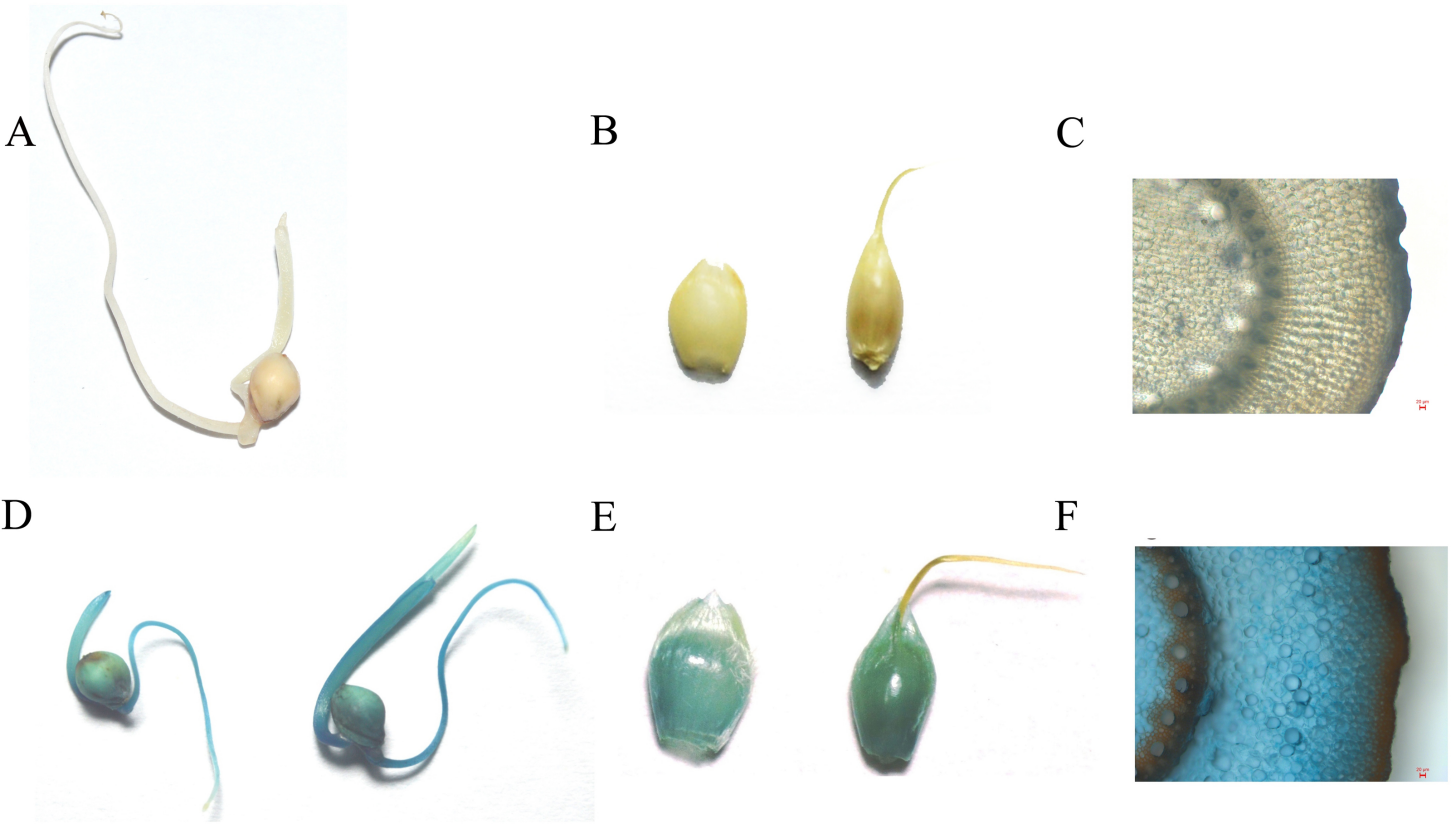
The GUS staining on control and transgenic T_1_ generation (A, B, C is control and C, D, E is T_1_ transgenic plant; A and C is seedlings; B and D is panicles; C and E is Stem, scale bar = 20 µm).

The analysis of GUS expression was performed on T_1_ plants to demonstrate transgene functionality in this transformant generation. The spikelets, seeds, and stems were selected for GUS staining (Fig. 5). The results illustrated that the *gus* gene was inherited and remained fully functional in the T_1_ generation.

### Phenotypic evulation of the transformant lines

No phenotypic differences were observed between transgenic plants and wild-type plants under greenhouse condition. The plant height, stem diameter, tiller number and seed yields were evaluated in triplicate between non-transformed wild-type plants and the transgenic lines (Table S1). Together, this analysis showed that there was no significant difference between wild-type plants and the T_1_ generation of transformant lines.

## Discussion

Since the first transgenic sorghum was reported in 1993, sorghum has been considered as a recalcitrant crop for genetic manipulation (Casas et al., 1993). In 2012, Liu and Godwin reported a highly improved sorghum transformation efficiency using Tx430 immature embryo as the transformation explant material . Since this report (Liu & Godwin, 2012), a series of research has been reported on sorghum transformation using different methods and sorghum accessions (Belide et al., 2017; Che et al., 2018; Liu et al., 2020). The use of immature embryo as the explant material has dominated these reports. However, the continuous supply of high-quality immature embryos for transformation is a labor-intensive, time-consuming, and expensive process (Belide et al., 2017). Besides, transformation efficiencies are influenced by environmental conditions such as light intensity and temperature (Do et al., 2016). Here, we report an efficient sorghum transformation protocol using mature seeds as the explant material for particle bombardment transformation. Compared to immature embryos, the use of mature seeds will significantly reduce the cost and labor of sorghum transformation. More importantly, as the explant material for tissue culture, mature seeds will provide more flexibility than the use of immature embryos.

The transformation efficiency is an important index for a transformation system. Previously, Liu & Godwin (2012) reported an average transformation efficiency of 20.7% was achieved *via* the use of particle bombardment and Tx430 immature embryos as the explant material. Later on, Wu et al. (2014) published that the highest efficiency of *Agrobacterium*-mediated transformation at 33% was obtained using the *Agrobacterium* strain AGL1 and Tx430 immature embryos as the explant material (Wu et al., 2014). Here, the best transformation efficiency of 18.75% was achieved with an average transformation rate of 13.33% obtained. Compared to previous reports (Liu & Godwin, 2012; Wu et al., 2014) , the transformation efficiency is slightly low but still competitive. To the best of our knowledge, no report exists on the successful transformation of sorghum using mature seeds as the explant material. Therefore, our system reported here provides an alternative sorghum transformation system for researchers.

The transformation efficiency is affected by many factors including the composition of the medium, the bombardment parameters, the plant expression vector used, and the strength of the constitutive promoter used to drive transgene expression (Casu, Selivanova & Perroux, 2012; Taparia, Gallo & Altpeter, 2012; Wu et al., 2014; Belide et al., 2017). Nirwan & Kothari (2003) and Liu, Gilding & Godwin (2013) identified that high levels of copper in the CIM and regeneration medium can enhance the callus induction rate and regeneration frequency. Based on previous reports, we optimized the concentration of copper sulfate in the regeneration medium for calli derived from mature seeds. The optimal media significantly increased the callus induction rate and regeneration rate (Li et al., 2017).

The DNA delivery parameters also play an important role in genetic transformation. Liu & Godwin (2012) reported that the optimization of bombardment parameters significantly increased the transformation frequency of sorghum (Liu & Godwin, 2012). Here, we compared three parameters including (1) helium pressure, (2) target distance, and (3) the diameter of the gold particle. The percentage of calli displaying GUS expression varied from 22.34 to 92.59%. This result showed that the optimization of bombard parameters is necessary and has a significant impact on transformation efficiency.

Transgenic plants with a single gene copy are important for investigating gene function as it reduces complexity of transgene expression. Transgenic production, only containing a single copy of target gene, is also highly valuable for crop commercialization because it is the most accepted form of transgene integration for subsequent regulation (Wu et al., 2014). The single gene copy in transgenic events was identified in present study. The target gene copy was detected by RT-PCR. Among 19 transgenic events, 21% of the transformant lines had a single transgene copy. A similar result was demonstrated in the previous report. Around a quarter of transgenic lines were confirmed as a single gene copy event by Southern blot hybridization (Liu & Godwin, 2012).

Genetic engineering, such as gene transformation and genome editing, plays an emerging role in modern agriculture. The genetic transformation system using mature seeds will notably reduce time, labor, and cost. Mature seeds as the explant material for sorghum transformation is not limited by the factors of time or weather. The present study will promote more research on sorghum genetic engineering.

##  Supplemental Information

10.7717/peerj.11849/supp-1 Supplemental Information 1 The phenotype comparison between wild type and T_1_ generation

10.7717/peerj.11849/supp-2 Supplemental Information 2 Correlation for APRT and TST

## Additional Information and Declarations

### Data Availability

The following information was supplied regarding data availability: Raw data are available as a Supplementary File.
