# Supplementary material for: An efficient sorghum transformation system using embryogenic calli derived from mature seeds
Source: PeerJ. 2021 Aug 5;9:e11849. doi: 10.7717/peerj.11849 (PMC8349514; doi:10.7717/peerj.11849)
Supplement: Supplemental Information 1 [file peerj-09-11849-s001.docx]

Supplementary table 1 The phenotype comparison between wild type and T_1_ generation

| Traits | Plant height  （cm） | Tiller number  (No.) | Stem diameter  (mm) | Fresh weight  (kg) | Seed yields (g) |
| --- | --- | --- | --- | --- | --- |
| Wild type | 324.6±13.6 | 0±0 | 15.14±1.45 | 1.05±0.15 | 50.3±5.79 |
| T1 generation | 326.7±15.3 | 0±0 | 15.41±0.97 | 1.02±0.09 | 47.9±3.59 |
